# A probability prediction method for the classification of surrounding rock quality of tunnels with incomplete data using Bayesian networks

**DOI:** 10.1038/s41598-022-19301-6

**Published:** 2022-11-18

**Authors:** Junjie Ma, Tianbin Li, Xiang Li, Shuanglong Zhou, Chunchi Ma, Daqiang Wei, Kunkun Dai

**Affiliations:** 1 College of Environment and Civil Engineering, Chengdu University of Technology, Chengdu, 610059 China; 2 State Key Laboratory of Geohazard Prevention and Geoenvironment Protection, Chengdu University of Technology, Chengdu, 610059 China

**Keywords:** Geology, Engineering, Civil engineering

## Abstract

The classification of surrounding rock quality is critical for the dynamic construction and design of tunnels. However, obtaining complete parameters for predicting the surrounding rock grades is always challenging in complex tunnel geological environment. In this study, a new method based on Bayesian networks is proposed to predict the probability for the classification of surrounding rock quality of tunnel with incomplete data. A database is collected with 286 cases in 10 tunnels, involving nine parameters: rock hardness, weathering degree, rock mass integrity, rock mass structure, structural plane integrity, in-situ stress, groundwater, rock basic quality, and surrounding rock level. Moreover, the Bayesian network structure is built using the collected database and quantitatively verified by strength analysis. Then, the accuracy, precision, recall, F-measure and receiver operating characteristic (ROC) curves are utilized for model evaluation. The average values of accuracy, precision, recall, F-measure, and area under the curve (AUC) are approximately 89.2%, 91%, 92%, 91%, and 0.98, respectively. These results indicate that the established classification model has high accuracy, even with small sample size and imbalanced samples. Ten additional sets of tunnel cases (incomplete data) are also used for verification. The results reveal that compared with the traditional Q-system (*Q*) and rock mass rating (*RMR*) classification methods, the proposed classification model has the lowest error rate and is capable of using incomplete data to predict sample results. Finally, sensitivity analysis suggests that the rock hardness and rock mass integrity have the strongest impact on the quality of tunnel surrounding rock. Overall, the findings of this study can serve as a useful reference for future rock mass quality evaluation in tunnels, underground powerhouses, slopes, etc.

## Introduction

Rock mass classification is widely used for assessing rock quality and performance based on the inherent structural parameters. According to the nature and requirements of engineering applications, the mechanical and geological properties of the rock mass are comprehensively considered in the classification of its quality^1^. In tunnel engineering, this process is called the classification of tunnel surrounding rock quality, which is one of the most important methods to evaluate the stability of surrounding rocks of tunnels, and it also plays a crucial role in the optimization of tunnel construction plan and design parameters. Since the 1920s, rock mass quality classification has received considerable research attention, and numerous classification methods have been proposed^2–9^. Table 1 lists several internationally recognized classification methods. Since the twenty-first century has witnessed a rapid development of underground engineering, especially the construction of a large number of deep-buried and ultra-long tunnels, the challenges encountered in the classification of tunnel surrounding rock quality must be urgently resolved. Therefore, it is necessary to further test the applicability of commonly used methods in tunnel construction and to develop new interdisciplinary methods for predicting the quality of surrounding rocks in tunnels and other underground projects.

**Table 1:** Rock mass quality classification methods.

Method	Origin	Indictors
*RSR*	Wickham et al.^5^	Rock type, rock hardness, geologic structure, joint spacing, joint attitude, direction of tunnel drive, groundwater
*RMR*	Bieniawski^6^	Rock strength, rock quality designation (*RQD*), joint spacing, joint state, groundwater, relationship between joint attitude and tunnel axis
*Q*	Barton^10^	*RQD*, joint set number, joint roughness number, joint alteration number, joint water reduction factor, stress reduction factor
*RMi*	Palmstrøm^8^	Uniaxial compressive strength of rock, joint state
*GSI*	Hoek and Brown^11^	Rock mass structure, structural surface characteristics
*HC*	Gong et al. ^12^	Rock strength, rock mass integrity, groundwater, state of main structural plane, relationship between structural plane attitude and tunnel axis
[*BQ*]	Chen et al.^13^	Rock strength, rock mass integrity, groundwater, in-situ stress, relationship between weak structural plane attitude and tunnel axis

The predictive classification of tunnel surrounding rock quality can be divided into long-term and short-term prediction. The long-term prediction aims to assess the quality of surrounding rock in the initial tunnel design stage and provide a reference for the preliminary design of tunnel^14^. It is generally used to provide a preliminary assessment of the basic quality (*BQ*) of rock mass based on its strength and integrity. The short-term prediction aims to evaluate the quality of surrounding rock in real-time based on various geological information revealed by tunnel excavation, which can serve as a reference for the dynamic design of tunnel support^14^. This study focuses on short-term classification prediction of tunnel surrounding rock quality, and it also utilizes long-term prediction.

Artificial intelligence (AI) was proposed at the Dartmouth Conference in 1956. Since then, it has been widely used in civil engineering applications, such as the prediction of pile bearing capacities^15^, peak particle velocity^16^, tunnel boring machine (TBM) penetration rate^17^, safety factor^18^, rock tensile strength^19^, Schmidt hammer rebound numbers^20^, and compressive strength of mortars^21^. Further, different AI algorithms have been employed for the prediction of rock mass classification, such as fuzzy mathematics^22,23^, neural networks^24,25^, support vector machines^26,27^, and random forest^28^. However, most of the existing intelligent rock mass classification methods focus on the construction stage (short-term prediction of rock mass quality), while the design stage is often ignored (long-term prediction of rock mass quality). More importantly, these existing methods require complete rock mass parameters to predict the rock mass quality. Due to the complex geological environment at the tunnel construction site, it is difficult to obtain the complete surrounding rock parameters. Therefore, it is imperative to further verify the applicability of the existing intelligent classification methods for surrounding rock quality.

The main problem in using the existing methods to predict the quality of tunnel surrounding rock is the difficulty in obtaining the indicators and missing data. Further, the geological information revealed by underground engineering excavation has great uncertainty. To solve this problem, Bayesian networks (BNs) have been introduced in intelligent surrounding rock classification (SRC). The advantage of BNs is that they can handle the conditional dependence between observed or unobserved random variables in the statistical model and can provide accurate probabilistic predictions based on a limited dataset. Therefore, BNs are widely used in pattern recognition, classification, and decision-making problems^29^. Furthermore, they are becoming increasingly popular in environmental science^29^, medicine^30,31^, agriculture^32^, and industrial fields^33,34^, and they are also being used in rock mechanics and rock engineering^35–38^. Notably, to establish a BN model, it is crucial to determine a reasonable BN structure. However, in the existing studies on BNs, the network structure is automatically generated by software or established by qualitative analysis. Therefore, it is necessary to quantitatively analyze the rationality of the network structure in future research.

**Figure 1:**
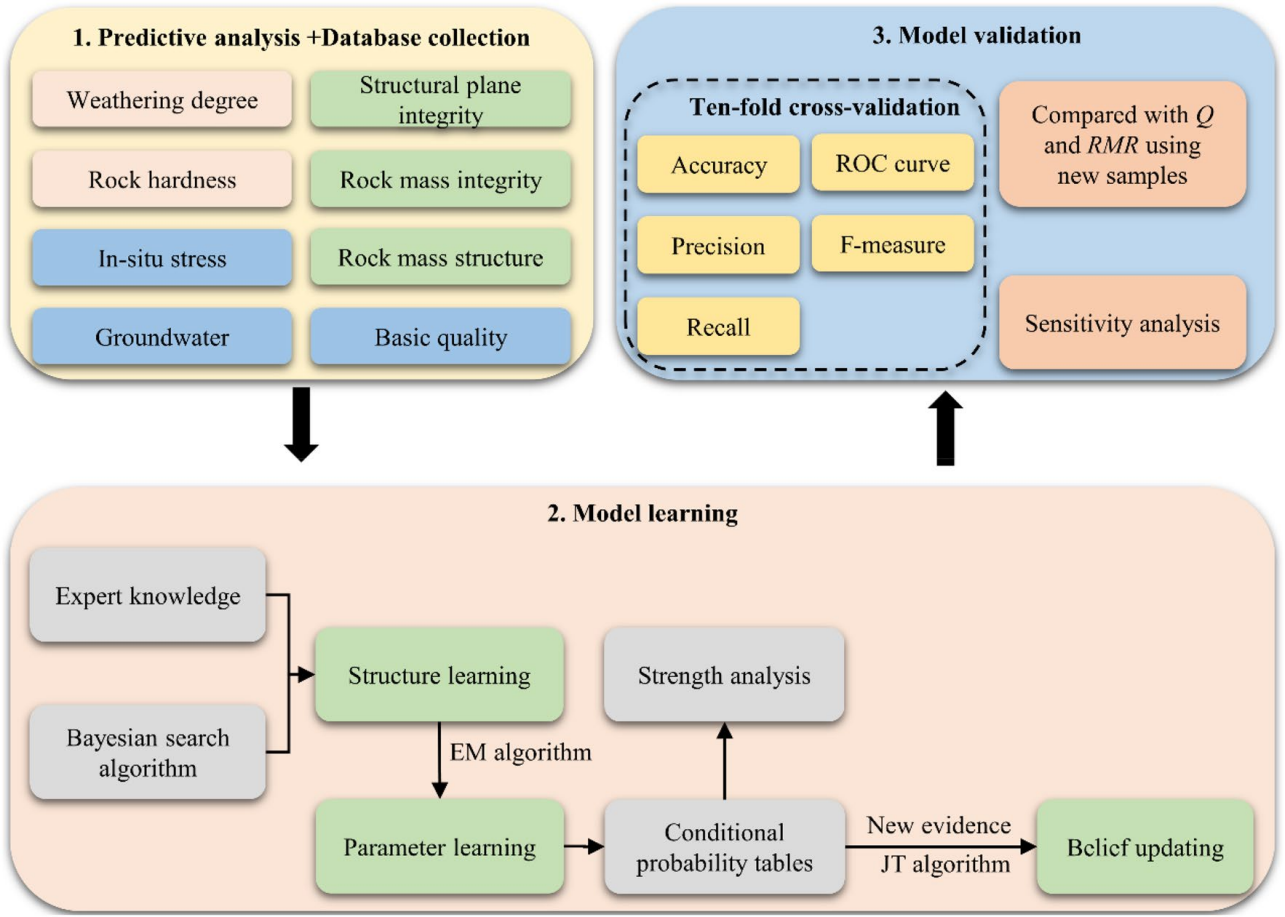
Workflow of the SRC-BN model.

For our analysis, we have compiled a case database containing 286 sets of data from 10 different tunnels with nine parameters: rock hardness, weathering degree, rock mass integrity, rock mass structure, structural plane integrity, in-situ stress, groundwater, rock basic quality, and surrounding rock level. Then, Bayesian search and expectation–maximization (EM) algorithms are used to learn the (SRC-BN) structure and parameters, respectively. Moreover, strength analysis is utilized to quantify the rationality of the learned SRC-BN structure. In addition, we examine and revise the learned conditional probability tables (CPTs) based on the expert experience, thereby improving the performance of the SRC-BN model in the case of small sample size and imbalanced samples. Finally, ten-fold cross-validation method and confusion matrix (including accuracy, precision, recall, F-measure, and receiver operating characteristic (ROC) curves) are used to evaluate the performance of the model. Furthermore, ten sets of other underground engineering cases are used to verify the feasibility of proposed SRC-BN model, and sensitivity analysis is employed to evaluate the importance of each input parameter in the model. The specific workflow of this study is shown in Fig. 1. The SRC-BN model can probabilistically predict the quality grade of tunnel surrounding rock with incomplete data. Moreover, the SRC-BN model overcomes the influence of small and unbalanced samples on the accuracy of SRC. In general, this probabilistic method for predicting rock mass quality grades is of immense significance for tunnel and underground engineering applications under the condition of large buried depth and high in-situ stress.

## Predictive analysis and data set description

### Inputs of SRC-BN

**Figure 2:**
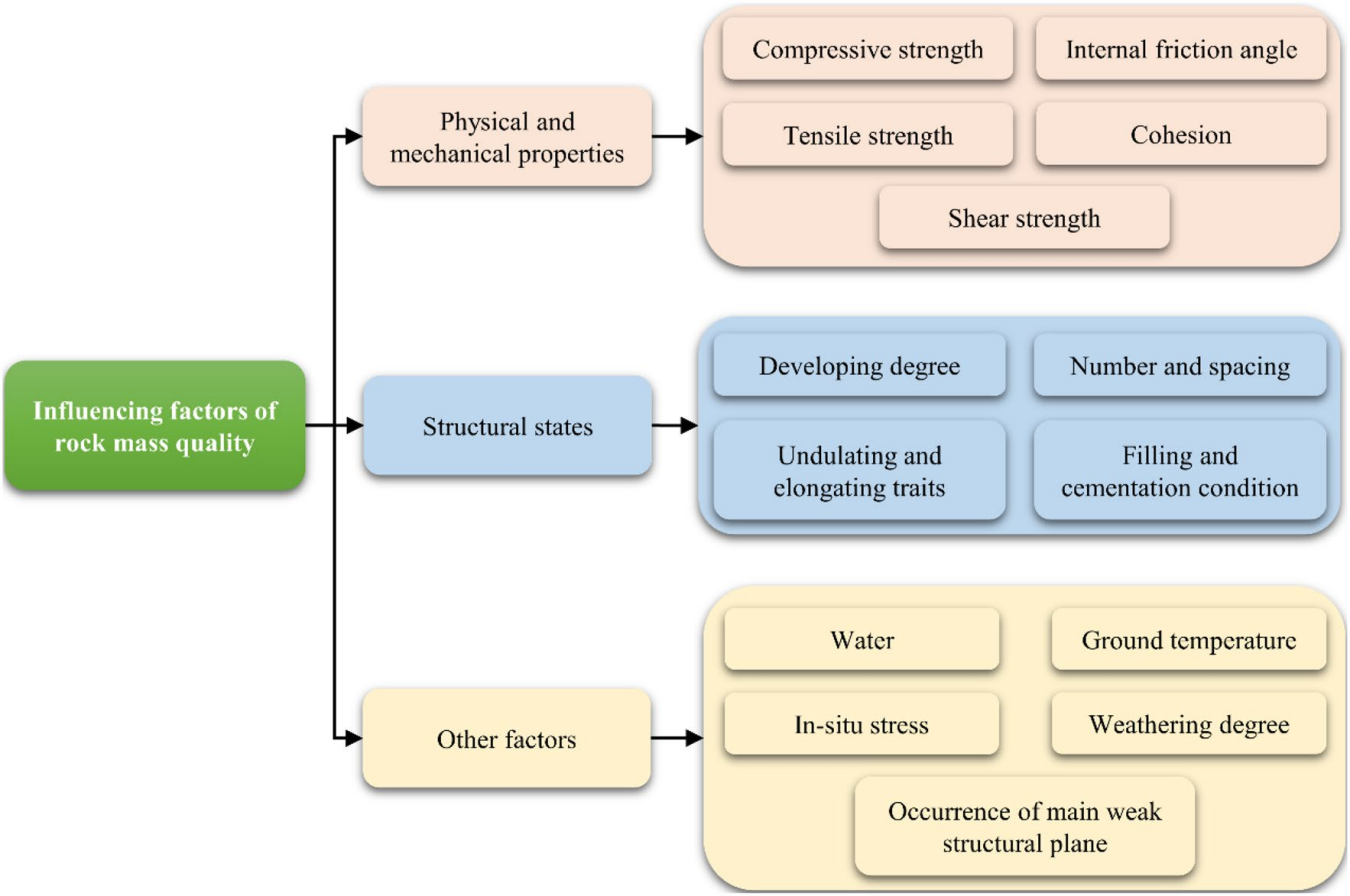
Classification of factors affecting rock mass quality.

Since 1920, numerous methods have been proposed for rock mass classification^39^. The internationally acceptable methods for evaluating the rock mass quality include rock structure rating (*RSR*), rock mass rating (*RMR*), Q-system (*Q*), geological strength index (*GSI*), and modified basic quality ([*BQ*]) classification methods, etc. The indicators of these classification methods are shown in Table 1. According to the existing studies on rock mass quality classification, the main influencing factors can be divided into physical and mechanical properties of rock, rock mass structural states, and other geological environment factors, as shown in Fig. 2.

Tunnel excavation is a dynamic process, and the changes in underground geological conditions are also uncertain. Therefore, the quality evaluation of tunnel surrounding rock is a dynamic probabilistic problem. BN is a powerful method to dynamically deal with datasets containing multiple variables and missing data as well as with conditional dependencies among the variables. It can also be used to quickly calculate the posterior probability of each state of the target node. It can be seen from Table 1 and Fig. 2 that the main factors affecting the surrounding rock quality of tunnels are rock hardness, rock integrity, in-situ stress, and groundwater. Hence, based on previous work^14^, we consider seven parameters that have a direct or indirect impact on the tunnel surrounding rock quality evaluation: weathering degree, rock hardness, structural plane integrity, rock mass structure, rock mass integrity, in-situ stress, and groundwater. Another parameter called *BQ* is used as an intermediate variable or as a categorical variable in the network. A brief description of these parameters and their acquisition methods are presented in the following subsections.

#### Rock hardness

Rock hardness is an important indicator to measure the quality of rock mass. The harder the rock, the better the rock quality^40^. The rock hardness can be measured by qualitative or quantitative methods, as shown in Table 2. The qualitative assessment is mainly based on the sound and rebound of geological hammer hitting the rock and the rock condition after immersion. The quantitative measurement of rock hardness is based on the saturated uniaxial compressive strength (*R*_c_) of rock. *R*_c_ can be obtained by uniaxial compressive strength test. The point load strength or rebound value of the rock can also be measured on site, and finally *R*_c_ is calculated by using empirical formula^41,42^. We recommend using on-site point load test or rebound test to obtain the *R*_c_ value indirectly and quantitatively.

**Table 2:** Qualitative and quantitative categorization of rock hardness.

Hardness	Qualitative characteristics	*R*_c_ (MPa)
Hard	The hammering sound is crisp with rebound, shaking hands, harder to break; after soaking in water, usually there is no water absorption reaction	(60, 250]
Slightly hard	The hammering sound is slightly crisp with slightly rebound, slightly shaking hands, hard to break; after soaking in water, slight water absorption reaction	(30, 60]
Slightly soft	The hammering sound is not clear and crisp, no rebound, easy to break; after soaking in water, nails can be imprinted	(15, 30]
Soft	The hammering sound is dumb, no rebound, dents, easier to break; after soaking in water, the hand can be broken	(5, 15]
Extremely soft	The hammering sound is dumb, no rebound, deep dents, and the hands can be crushed; after soaking in water, it can be crushed into a ball	(0, 5]

#### Weathering degree

The weathering degree refers to the degree of damage to the rock by weathering. The higher the weathering degree, the lower the rock strength. The weathering degree is also often used to classify the surrounding rock quality of tunnels^43^. The qualitative and quantitative classification criteria of rock weathering degree are shown in Table 3. Qualitative classification is a comprehensive judgment based on the characteristics of rock structure, mineral composition, degree of fragmentation, etc. Quantitative classification is based on the wave velocity ratio (*k*_*r*_) or weathering coefficient (*k*_*f*_) of the rock^44^. *k*_*r*_ refers to the ratio of compressional wave velocity between weathered rock and fresh rock. *k*_*f*_ refers to the ratio of the saturated uniaxial compressive strength between weathered rock and fresh rock.

**Table 3:** Qualitative and quantitative classification of rock weathering degree.

Degree	Qualitative characteristics	*k*_*r*_	*k*_*f*_
Fresh	The rock structure does not change, and the rock quality is fresh	(0.9, 1.0]	(0.9, 1.0]
Slight	The rock structure, mineral composition, and color are basically unchanged, and some fracture surfaces contain iron and manganese or are slightly discolored	(0.8, 0.9]	(0.8, 0.9]
Medium	The rock structure is partially destroyed; the mineral composition and color are obviously changed, and the fracture surface is severely weathered	(0.6, 0.8]	(0.4, 0.8]
Severe	Most of the rock structure is destroyed, and the mineral composition and color are obviously changed. The feldspar, mica, and iron-magnesium minerals are weathered and altered	(0.4, 0.6]	(0, 0.4]
Extreme	The rock structure is completely destroyed, disintegrated, and decomposed into loose soil or sand. All minerals are discolored, and the luster disappears. Most of the minerals except quartz particles are weathered and eroded into secondary minerals	[0.2, 0.4]	–

#### Rock mass integrity

The rock mass integrity is one of the most important parameters that affect the tunnel surrounding rock quality^45^. The rock mass integrity reflects the development of structural planes, including the number of joint sets, joint spacing, and the degree of combination of main structural planes that affect the stability of the surrounding rock. The quantitative classification of rock mass integrity is shown in Table 4. This classification is based on the rock mass integrity coefficient (*K*_v_). *K*_v_ is the square of the ratio of elastic longitudinal wave velocity (*V*_pm_) of rock mass to the elastic longitudinal wave velocity (*V*_pr_) of rock (block)^44^. In actual engineering, it is more common to measure the volume joint number (*J*_v_) of the rock mass to obtain the *K*_v_ value and finally obtain the rock mass integrity. It is worth noting that the unit of *J*_v_ is number of joints per m^3^.

**Table 4:** Quantitative classification of rock mass integrity.

Rank	Complete	Slightly complete	Slightly broken	Broken	Extremely broken
*J*_v_	[0, 3)	[3, 10)	[10, 20)	[20, 35)	[35, + ∞)
*K*_v_	(0.75, 1]	(0.55, 0.75]	(0.35, 0.55]	(0.15, 0.35]	(0, 0.15]

#### Rock mass structure

The rock mass structure is composed of structural plane and structural body, which reflects the development degree of structural plane and the fragmentation of rock mass. The structural plane is divided into weak plane surface and hard structural plane. The structural body is divided into block shape and plate shape according to the mechanical action. Structural plane and body are combined and arranged differently in the rock mass to form different types of structures. The rock mass structure can be comprehensively judged according to the rock mass integrity, characteristics of rock mass, and development of structural plane. Determination of the type of rock mass structure plays an important role in evaluating the stability of the rock mass under engineering loads. The Chinese hydropower industry has proposed the classification and semi-quantitative identification methods of rock mass structure (see Table 5)^44^.

**Table 5:** Classification of rock mass structure.

Type	Subtype	Characteristics of rock mass structure
Block structure	Integral structure	The rock mass is complete with huge-block shape and undeveloped structural plane, and the spacing is greater than 1 m
	Block structure	The rock mass is slightly complete with block shape and slightly developed structural plane, and the spacing is generally 1–0.5 cm
	Fractured block structure	The rock mass is slightly complete with fractured block shape and moderately developed structural plane, and the spacing is generally 0.5–0.3 m
Layered structure	Extremely-thick layered structure	The rock mass is complete with extremely-thick layered shape and no developed layers, and the spacing is greater than 1 m
	Thick layered structure	The rock mass is slightly complete with thick layered shape and slightly developed layers, and the spacing is generally 1–0.5 cm
	Medium-thick layered structure	The rock mass is slightly complete with medium-thick layered shape and moderately developed layers, and the spacing is generally 0.5–0.3 cm
	Interlayer structure	The rock mass is slightly complete or has poor integrity with inter-layered shape and slightly developed or developed layers, and the spacing is generally 0.3–0.1 m
	Thin layered structure	The rock mass has poor integrity with thin-layered shape and developed layers, and the spacing is generally less than 0.1 m
Mosaic structure		The rock mass integrity is poor, the rock blocks are tightly embedded, and the structural plane is slightly developed to developed. The spacing is generally 0.3–0.1 m
Fragmented structure		The rock mass is broken, the structural plane is well developed, and the spacing is generally less than 0.1 m
Granular structure		The rock mass is broken. Rocks, cuttings, and mud are mixed together

#### Structural plane integrity

Structural plane integrity is comprehensively assessed according to the opening degree, roughness, and filling of the structural plane. It reflects the characteristics of the structural surface, including the degree of opening and filling, and it is closely related to the rock mass integrity. Rock mass with good integrity usually has a better degree of structural plane integration. The Ministry of Water Resources of the People’s Republic of China (2014) proposed a semi-quantitative classification standard for the structural plane integrity^44^, as shown in Table 6.

**Table 6:** Classification and characteristics of the structural plane integrity.

Integration degree	Characteristics
Good	(1) The opening degree of structural plane is less than 1 mm, and the filling is siliceous, iron, or calcium cement. Otherwise, the structure plane is rough and there is no filling (2) The opening degree of structural plane is 1–3 mm, and the filling is siliceous or iron cement (3) The structural plane is rough and the opening degree is greater than 3 mm, and the filling is siliceous cement
Ordinary	(1) The structural plane is straight and the opening degree is less than 1 mm, and the filling material is cement with calcareous mud or there is no filling material (2) The opening degree of the structural plane is 1–3 mm, and the filling is calcium cement (3) The structural plane is rough and the opening degree is greater than 3 mm, and the filling is iron or calcium cement
Bad	(1) The structural plane is straight and the opening degree is 1–3 mm, and the filling is argillaceous cement or calcium argillaceous cement (2) The opening degree of structural plane is greater than 3 mm, and the filling is mostly muddy or rock debris
Very bad	The filling is muddy or mud with rock cuttings, and the thickness is greater than the undulation difference

#### In-situ stress

In-situ stress is the stress confined in a rock formation before excavations or other perturbations. It is an important mechanical property, which affects the bearing capacity of rock mass. For rock mass existing in a certain in-situ stress environment, the greater the confining pressure formed by in-situ stress, the greater the bearing capacity of rock mass^46^. At the same time, under high in-situ stress conditions, hard rocks are prone to rockburst disasters, while soft rocks are prone to large deformation disasters^47,48^. Therefore, the in-situ stress has a significant influence on the quality of tunnel surrounding rock. The internationally popular *Q* and [*BQ*] methods consider the correction of in-situ stress to evaluate the surrounding rock quality. The quantitative classification of in-situ stress is shown Table 7. Here, *σ*_max_ is the maximum initial stress perpendicular to the hole axis.

**Table 7:** Classification of in-situ stress.

Level	Low	Medium	High	Extremely high
*R*_c_/*σ*_max_	[9, + ∞)	[7, 9)	[4, 7)	(0, 4)

#### Groundwater

As an important environmental factor, groundwater affects the deformation and destruction of rock mass, thereby influencing its stability. Expansive soft rock is a disaster for underground engineering, and its mechanical properties depend on the degree of interaction between soft rock and water. It can be seen from Table 1 that the methods such as *Q*, *RMR*, and [*BQ*] also consider the impact of groundwater on the surrounding rock quality. However, in most methods, the impact of groundwater on the surrounding rock quality is evaluated by quantifying the water pressure, which is often impossible in actual engineering applications. Therefore, based on engineering experience, a qualitative description of different water emergence conditions from the tunnel excavation surface is presented in Table 8, where *p* is the water pressure of surrounding rock fissures (MPa), and *q* is the water output per 10 m hole length (L/min∙10 m).

**Table 8:** Classification of water outflow from tunnel excavation face.

Type	Qualitative description	Quantitative division
Dry	Excavation face is completely dry	*p* ∈ [0, 0.01] or *q* ∈ [0, 5]
Wet	Wet marks can be seen in some areas of the excavation face	
Moist	Water can be seen or leached from the excavation face	*p* ∈ (0.01, 0.1] or *q* ∈ (5, 25]
Dripping	Water drips from the excavation face	
Rain-like dripping	There are drops of water resembling rain on the excavation face	*p* ∈ (0.1, 0.5] or *q* ∈ (25, 125]
Linear water	The excavation face has similar water pipes, and the water column is smaller (the water volume and water pressure are smaller)	
Tubular water	The excavation face has similar water pipes, and the water column is relatively thick (the water volume and water pressure are very large)	*p* ∈ (0.5, ∞) or *q* ∈ (125, ∞)
Gushing water	There is a large water outlet on the excavation face; the flow and velocity are large, which often causes great damage to the project	

#### Rock mass basic quality

The [*BQ*] method uses the hardness and the integrity of rock mass to make a preliminary assessment of its quality. The classification of *BQ* is shown in Table 9. The [*BQ*] method is widely used in China, where the determination of *BQ* value is the basis for the final quality assessment of tunnel surrounding rock. *BQ* is also crucial for the preliminary design of the initial support structure of tunnel. Generally, due to the urgency of tunnel construction, the strength and integrity of rock mass are not determined early enough at the construction site. In the tunnel design stage, the quality grade of tunnel surrounding rock is often judged preliminarily. When the strength and integrity of rock mass are not measured, the surrounding rock quality grade obtained from the tunnel design stage can be input as the *BQ* value into the model. In this way, more parameters can be input in the SRC-BN model to improve its reliability. Similarly, in the tunnel planning stage, the strength and integrity of rock mass can be approximated based on the geological information revealed by the borehole, and then the *BQ* value can be preliminary assessed using the proposed SRC-BN model.

**Table 9:** Classification of *BQ.*

Rank	5	4	3	2	1
Score	(0, 250]	(250, 350]	(350, 450]	(450, 550]	(550, 710]

### Description of database

**Figure 3:**
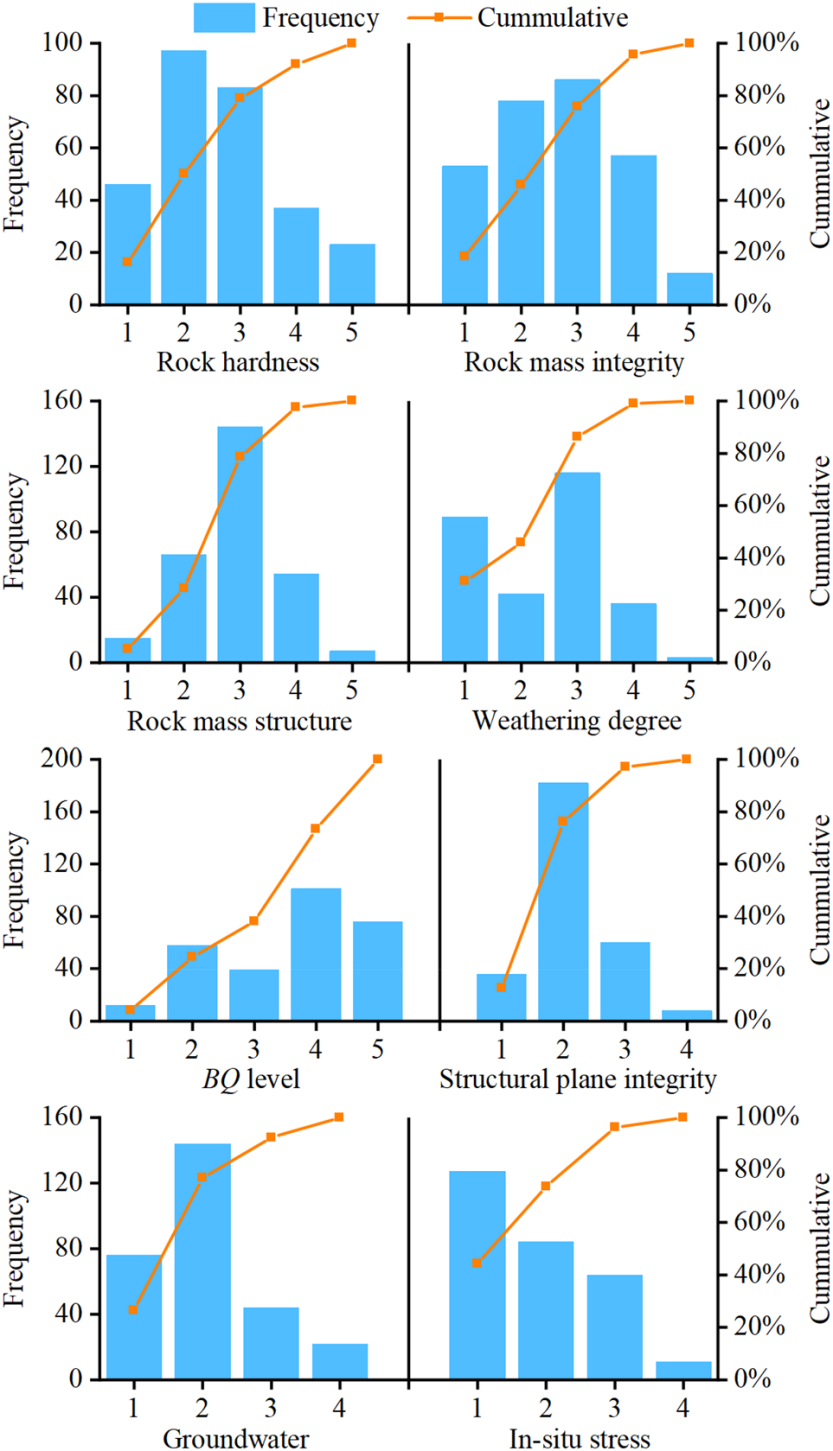
Histograms of the eight parameters considered to predict the quality of tunnel surrounding rock. The numbers on the X-axis represent the characteristic of different parameters. (1) The numbers 1, 2, 3, 4, and 5 for rock hardness represent hard, slightly hard, slightly soft, soft, and extremely soft, respectively. (2) 1, 2, 3, 4, and 5 for rock mass integrity represent complete, slightly complete, slightly broken, broken, and extremely broken, respectively. (3) 1, 2, 3, 4, and 5 for rock mass structure represent integral/extremely-thick layered, block/thick layered, fractured block/medium-thick layered/interlayer/thin layered/mosaic, fragmented, and granular, respectively. (4) 1, 2, 3, 4, and 5 for weathering degree represent fresh, slight, medium, severe, and extreme, respectively. (5) 1, 2, 3, and 4 for structural plane integrity represent good, ordinary, bad, and very bad, respectively. (6) 1, 2, 3, and 4 for groundwater represent dry/wet, moist/dripping, rain-like dripping/linear water, and tubular water/gushing water, respectively. (7) 1, 2, 3, and 4 for in-situ stress represent low, medium, high, and extremely high, respectively.

We have collected a tunnel surrounding rock quality evaluation database containing nine parameters: rock hardness, weathering degree, rock mass integrity, rock mass structure, structural plane integrity, in-situ stress, groundwater, *BQ* and surrounding rock level. In this database, *BQ* are expressed in terms of the quality grade and not the quality score of the surrounding rock. Surrounding rock level is the quality grade of surrounding rock referenced by the actual support at the tunnel construction site. In the construction of traffic tunnels in China, surrounding rock level is mainly determined by [*BQ*] method (see Appendix A for [*BQ*] method). The parameter *BQ* can be used as an intermediate or categorical parameter in the established SRC-BN structure. Most of tunnel surrounding rock quality evaluation cases have been collected from Niu^14^, and a few cases are from unpublished scientific reports. These data enabled us to compile a new database of tunnel surrounding rock quality, which is used in our analysis. The database has a total of 286 cases, which are from ten typical tunnels in China (Niba mountain tunnel, Zhegu mountain tunnel, Erlang mountain tunnel, Wangjiangling tunnel, Zaozilin tunnel, Longxi tunnel, Futang tunnel, Miyaluo #3 tunnel, Shiziping tunnel, and Micang mountain tunnel). Figure 3 shows the distribution of nine parameters in the 286 cases. Our database covers a wide range of values for these nine parameters. Therefore, in principle, it has a good applicability within the scope of considered cases. With the emergence and addition of new data in the future, this method is expected to become more generalized and practical.

### Parameters excluded in the SRC-BN model

Except the parameters mentioned in “Inputs of SRC-BN”, other parameters can also be used to predict the surrounding rock quality level of tunnels. However, we do not consider them in the SRC-BN model due to the following reasons:

#### RQD

*RQD* was proposed by Deere et al.^4^ to quantify the rock quality. The internationally used *Q* and *RMR* methods for the surrounding rock quality classification use *RQD* indicator. However, *RQD* has certain limitations. Firstly, a specific size of drill bit and coring equipment must be used to obtain the standard *RQD* value. Because cores are considered in weak and unintegrated rock masses, the *RQD* values obtained by using different drill bits and coring equipment vary greatly^49–51^. Secondly, *RQD* roughly counts cores with lengths greater than 10 cm and less than 10 cm^52^. Finally, since we have selected the index of rock mass integrity, the impact of rock mass completeness on the tunnel surrounding rock quality is considered. Therefore, *RQD* is not considered in the established SRC-BN model.

#### Occurrence of main weak structural plane

The relationship between the occurrence of main weak structural plane and the tunnel axis has a certain influence on the surrounding rock stability^53,54^. This parameter is a crucial indictor in the design of tunnel in the active area of neotectonic movement. This parameter is considered in the [*BQ*], *HC*, and *RMR* methods, but it is not considered in *Q*, *GSI*, and *RMi* methods. Since this parameter is also missing in the collected data samples, we did not consider it in the established SRC-BN model. In future research, we will consider including this parameter in the SRC-BN model.

#### Joint features

The joint characteristics include joint set number, joint spacing, joint length, joint roughness, and joint filling. Some joint features are considered in both *Q* and *RMR* methods. The five main joint features essentially reflect the structural characteristics and integrity of rock mass. In particular, the structure and integrity of rock mass have been considered in the established SRC-BN model. Joint roughness and joint filling basically reflect the completeness of structural plane. They are also indirectly considered in the established SRC-BN model. Hence, the five joint features are not employed in the SRC-BN model.

#### Other parameters

Traffic tunnels passing through areas with special geological structures may encounter distinct problems such as high ground temperature and karst. These geological issues should also be considered in the quality evaluation of tunnel surrounding rock. However, there is no case with such issues in the collected surrounding rock samples. Thus, these issues cannot be considered in the established SRC-BN model. Fortunately, we have collected some samples under high in-situ stress environment, so our model considers the impact of high in-situ stress on the tunnel surrounding rock quality. In subsequent studies, we will further consider the effect of special geological issues on the tunnel surrounding rock quality.

## Establishment of SRC-BN structure and parameter learning

### SRC-BN structure definition

BN model is probabilistic graphical model, which was proposed by Judea Pearl in 1986^55^. BN is a directed acyclic graph composed of nodes and arrows connecting nodes. The nodes in BN indicate random variables, and the arrows connecting nodes represent the relationship between two variables. This relationship is quantitatively expressed by conditional probability distributions. Therefore, there is no arrow between two conditionally independent variables^56^.

In the BN model, the network structure must be defined first to carefully consider the conditional independence and independent relations among the input variables. BN structure can be defined through structural learning^56^ or experience. After the BN structure is defined, the conditional probability between the variables must be defined. The CPTs can be used to represent the quantitative dependence among the discrete variables. When new evidence (observed variable) is introduced into the BN or the existing evidence is updated, the Bayesian method is used to calculate the changes in CPTs of unobserved variables. This process is called “belief updating,” which is described in “Belief updating”.

BN can be used to solve an uncertain problem, such as classification of tunnel surrounding rock quality. Here, ***Y*** is used to represent the class variable, which is divided into five levels (***Y*** = 1, 2, 3, 4, 5). ***X*** is used as an input vector, which includes *n* observed or unobserved input variables that affect the class variable ***Y*** (***X*** = (*X*_1_, *X*_2_, …, *X*_*n*_)). In this work, ***X*** is given by the seven input variables mentioned in “Inputs of SRC-BN”. In addition, the variable *BQ* can be used as an intermediate variable or a categorical variable. Subsequently, the collected 286 cases (mentioned in “Description of database”) are used to train the SRC-BN model.

**Figure 4:**
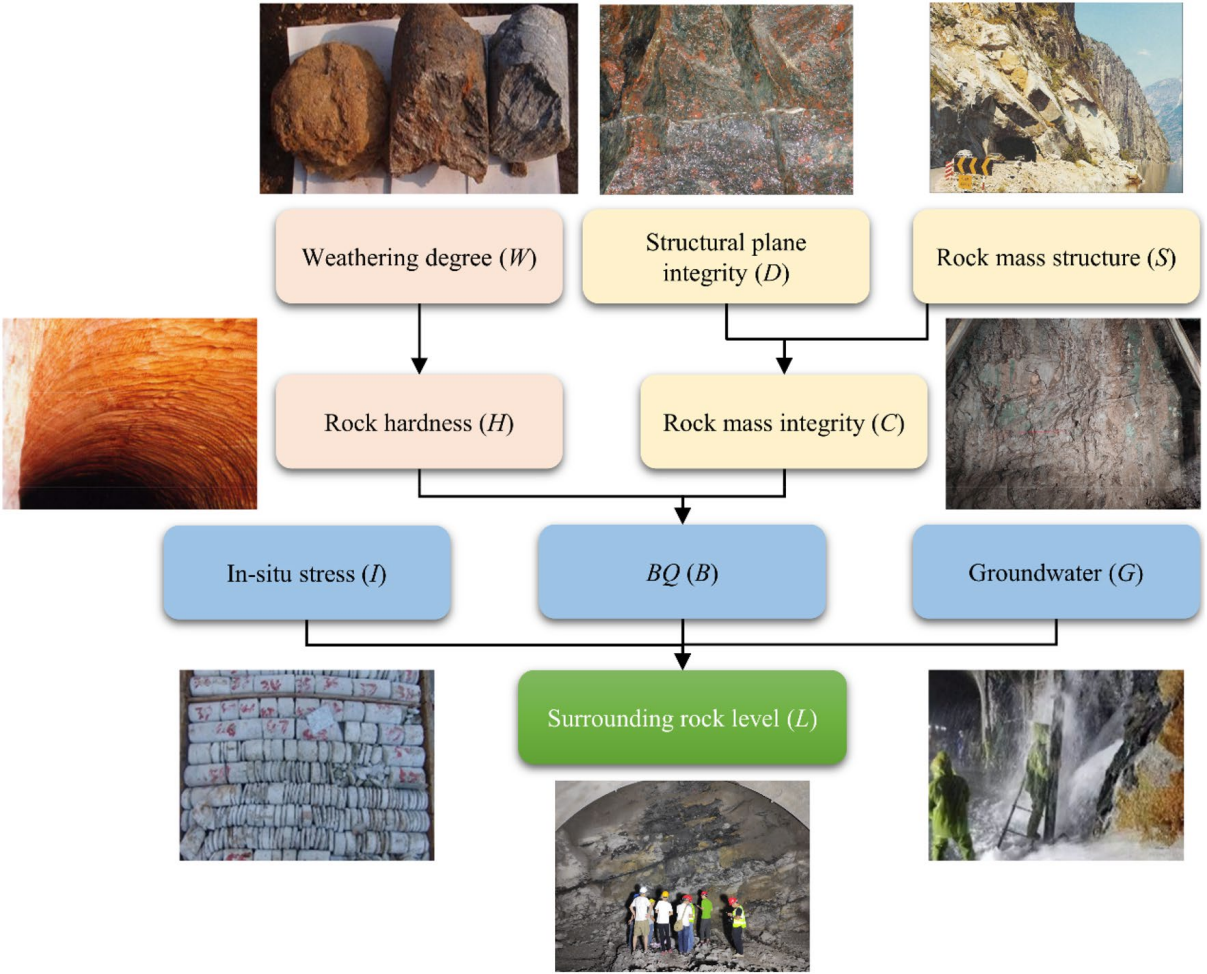
SRC-BN structure of tunnel surrounding rock quality.

A structural learning algorithm, called Bayesian Search, is used to establish the BN structure^56,57^. Before examining the BN structure, we are allowed to increase the professional background knowledge to obtain a reasonable network structure. Since the input parameters are qualitative indicators or discretized continuous indicators, the calculation of the statistical correlation between the input parameters has little reference value. Fig. 4 illustrates the SRC-BN structure for predictive classification of tunnel surrounding rock quality, given seven input parameters: weathering degree, rock hardness, structural plane integrity, rock mass structure, rock mass integrity, in-situ stress, and groundwater. In addition, *BQ* can be used as an intermediate parameter. It is especially useful when the rock hardness or rock mass integrity input parameters are missing.

### Discretization of parameters

Among the eight parameters, rock mass structure, structural plane integrity, and groundwater are the qualitative indicators (discrete variables), while the remaining five parameters are continuous variables. To use the continuous variables in BN, it is necessary to discretize them or specify a density function for them^58^. Under limited sample conditions, it is difficult to specify an accurate density function for the input parameters. Therefore, the discretization method is used to deal with continuous variables. The discretization method divides the continuous value into several ranges. In this article, we adopt the parameter division range recommended by the industry guide “*Standard for Engineering Classification of Rock Mass*.” The eight parameters have been discussed in “Inputs of SRC-BN”. The interval range and possible states of each node in SRC-BN are shown in Table 10.

**Table 10:** Intervals of the input parameters of SRC-BN.

Parameter	Intervals
**Rock hardness**	
Intervals [MPa]	(0, 5], (5, 15], (15, 30], (30, 60], (60, 250]
States	Hard, slightly hard, slightly soft, soft, extremely soft
**Weathering degree**	
Intervals [*k*_*r*_]	[0.2, 0.4], (0.4, 0.6], (0.6, 0.8], (0.8, 0.9], (0.9, 1]
States	Fresh, slight, medium, severe, extreme
**Rock mass integrity**	
Intervals [*K*_v_]	(0, 0.15], (0.15, 0.35], (0.35, 0.55], (0.55, 0.75], (0.75, 1]
States	Complete, slightly complete, slightly broken, broken, extremely broken
**Rock mass structure**	
States	Integral/extremely-thick layered, block/thick layered, fractured block/medium-thick layered/interlayer/thin layered/mosaic, fragmented, granular
**Structural plane integrity**	
States	Good, ordinary, bad, very bad
**In-situ stress**	
Intervals [*R*_c_/*σ*_max_]	[9, + ∞), [7, 9), [4, 7), (0, 4)
States	Low, medium, high, extremely high
**Groundwater**	
States	Dry/wet, moist/dripping, rain-like dripping/linear water, tubular water/gushing water
***BQ***	
Intervals	(0, 250], (250, 350], (350, 450], (450, 550], (550, 710]
States	5, 4, 3, 2, 1

### Learning parameters

**Figure 5:**
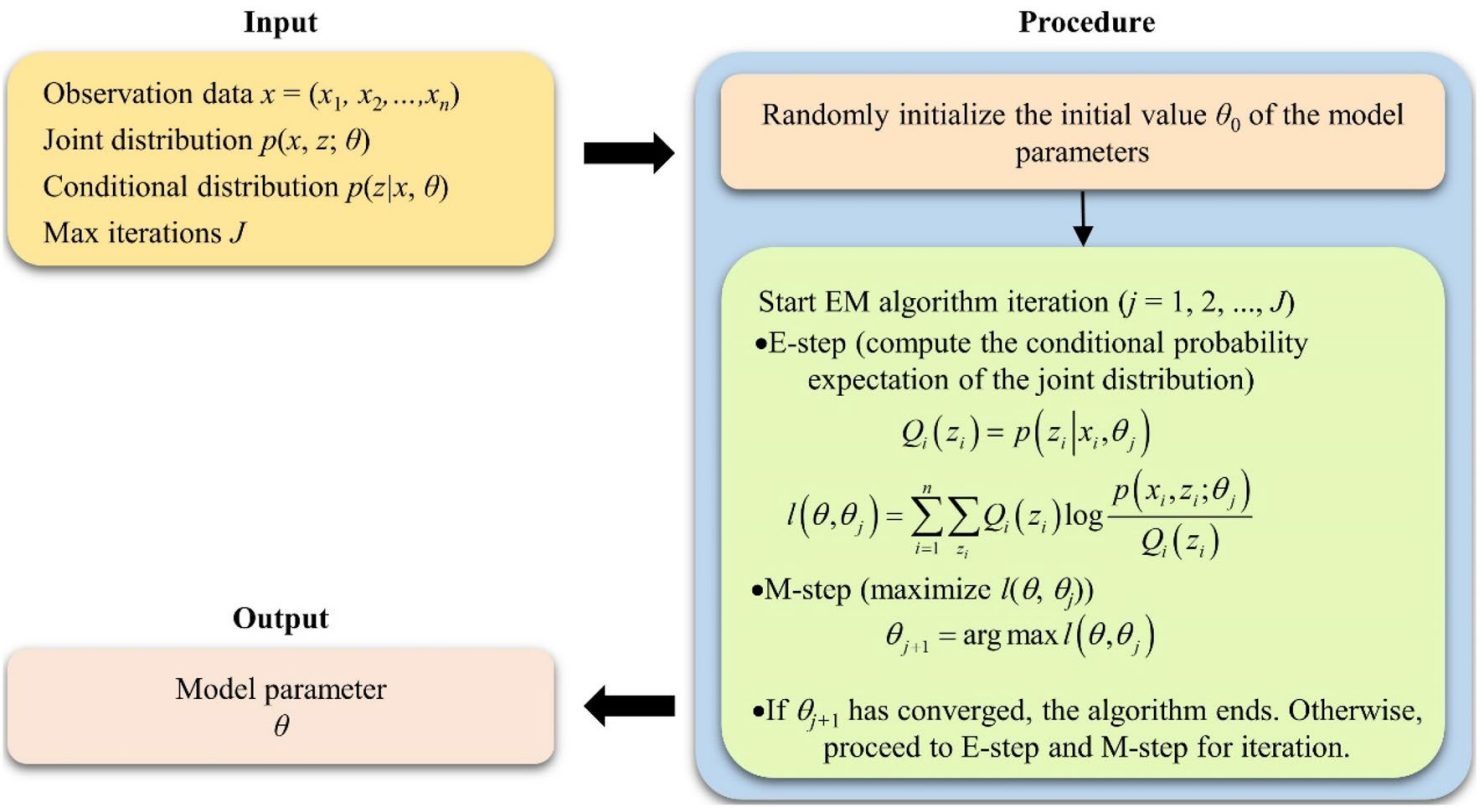
Workflow of the EM algorithm.

Based on the model structure defined in Fig. 4, the SRC-BN model can learn parameters from dataset. In other words, the CPTs of each node in the SRC-BN model can be obtained through training and learning. Generally, the expectation–maximization (EM) algorithm is used to estimate the CPTs of each node in the BN^59^. The EM algorithm can find the maximum likelihood estimate of a set of parameters even when some variables in the dataset are missing^58,60^. The workflow of the EM algorithm is show as Fig. 5.

The standard calculation framework of EM algorithm is composed of expectation-step (E-step) and maximization-step (M-step). E-step mainly estimates the parameters by observing data and existing models, and then this estimated parameter value is used to calculate the expected value of the likelihood function. M-Step is to find the corresponding parameter when the likelihood function is maximized. Further details about the EM algorithm can be found in Jensen and Nielsen^58^.

### Belief updating

**Figure 6:**
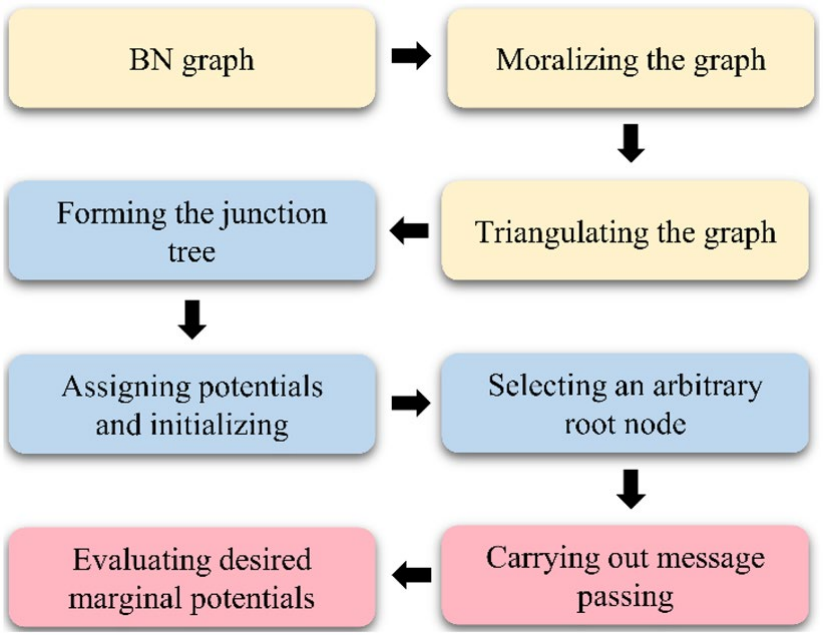
Workflow of the JT algorithm.

After obtaining SRC-BN parameters by using EM algorithm, belief updating is the next step. Belief updating is also called probabilistic inference, and it is used to calculate the posterior probability of a given evidence (observations)^60^. In particular, for predictive analysis of the tunnel surrounding rock quality, this method is used to compute the posterior probability of a given evidence, where the evidence may be a set of incomplete observations about the input parameter vector. Generally, the junction tree (JT) algorithm is used to calculate the posterior probability^61^. The workflow of the JT algorithm is show as Fig. 6.

The calculation steps of the JT algorithm in belief updating are summarized as follows: (1) a junction tree is constructed based on the existing BN; (2) a message passing algorithm is used to convey messages along the junction tree; (3) response to queries are provided when introducing evidence. Further details of the JT algorithm can be found in Jensen and Nielsen^58^ and Korb and Nicholson^60^.

### Strength analysis

The established SRC-BN structure is a tree structure. Therefore, we need to analyze the influence strength of parent node on the child node in this tree structure. Then, the result can be used to quantitatively judge the rationality of the SRC-BN structure (the relationship between the input parameters of SRC-BN are analyzed from a qualitative perspective in “Inputs of SRC-BN”, which is the expert background knowledge before the establishment of the SRC-BN structure). The influence strength is calculated based on the CPT of child node, which essentially expresses the distance between various conditional probability distributions over the child node conditional on the states of the parent node^62^. We use the Euclidean distance in GeNIe to calculate the influence strength^63^. Suppose *A* and *T* are two points in n-dimensional space, and their discrete probability distributions are shown in Eq. (1).

1 $$\left\{ {\begin{array}{*{20}l} {A \in \left\{ {\left( {a_{1} ,a_{2} , \ldots ,a_{n} } \right)\left| {a_{i} > 0,\sum\limits_{i = 0}^{n} {a_{i} = 1} } \right.} \right\},n > 1} \hfill \\ {T \in \left\{ {\left( {t_{1} ,t_{2} , \ldots ,t_{n} } \right)\left| {t_{i} > 0,\sum\limits_{i = 0}^{n} {t_{i} = 1} } \right.} \right\},n > 1} \hfill \\ \end{array} } \right.$$

The calculation of spatial distance (normalized) between two points is shown in Eq. (2).

2 $$E_{norm} \left( {A,T} \right) = \frac{{\sqrt {\sum\nolimits_{i = 1}^{n} {\left( {a_{i} - t_{i} } \right)^{2} } } }}{\sqrt 2 }$$

**Figure 7:**
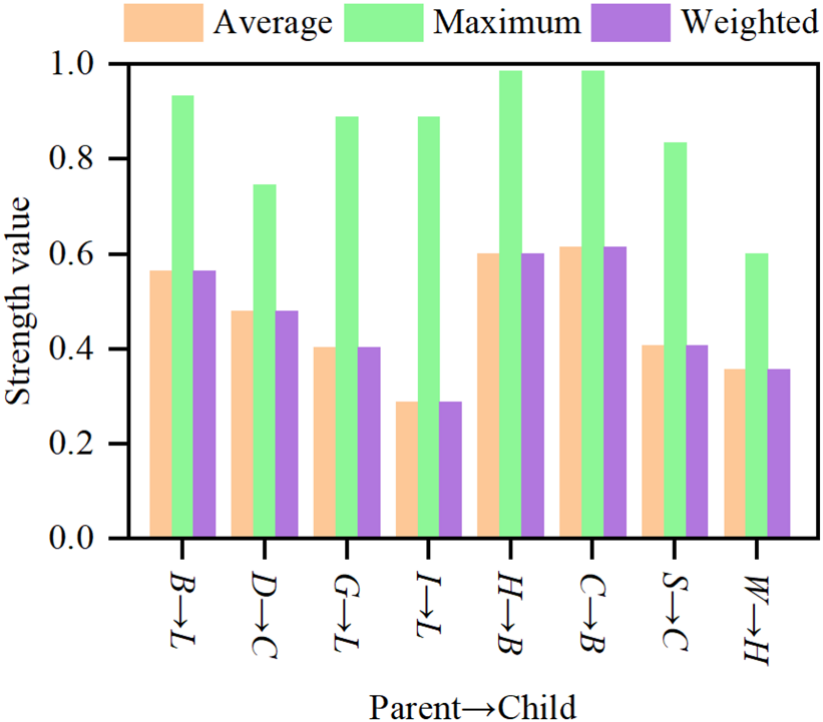
Results of strength analysis of the SRC-BN model.

Figure 7 shows the strength analysis results obtained using GeNIe. Average indicates the average value of the distance. Maximum is the largest distance from the distribution. Weighted is the edge probability of the parent node to measure the distance. We usually only focus on the average and maximum values. The larger the calculated value, the stronger the influence. According to Fig. 7, the following conclusions can be drawn: (1) the average value of node *H* (rock hardness) and node *C* (rock mass integrity) on node *B* (*BQ*) exceeds 0.6, and the maximum value is also above 0.9. From the description in “Inputs of SRC-BN”, it can be seen that *BQ* is mainly judged based on the hardness and integrity of rock mass. Therefore, the qualitative and quantitative assessments of the structure are consistent. (2) The average value of node *B* to node *L* (classification of tunnel surrounding rock quality) exceeds 0.5, and the maximum value also reaches above 0.9. According to the description in “Inputs of SRC-BN”, *BQ* is a preliminary judgment of the surrounding rock quality. In addition, the maximum value of node *G* (groundwater) and node *I* (in-situ stress) to node *L* are both above 0.8. Consequently, the qualitative (in “Inputs of SRC-BN”) and quantitative judgment results of the structure are coincident. (3) The quantitative calculation results of the tree structure at *D* → *C* (gomphosis → rock mass integrity), *S* → *C* (rock mass structure → rock mass integrity), and *W* → *H* (weathering → rock hardness) are also consistent with the expert background knowledge. Therefore, the established SRC-BN structure is reasonable from both qualitative and quantitative perspectives.

## Results and discussion

### Learned SRC-BN model

**Figure 8:**
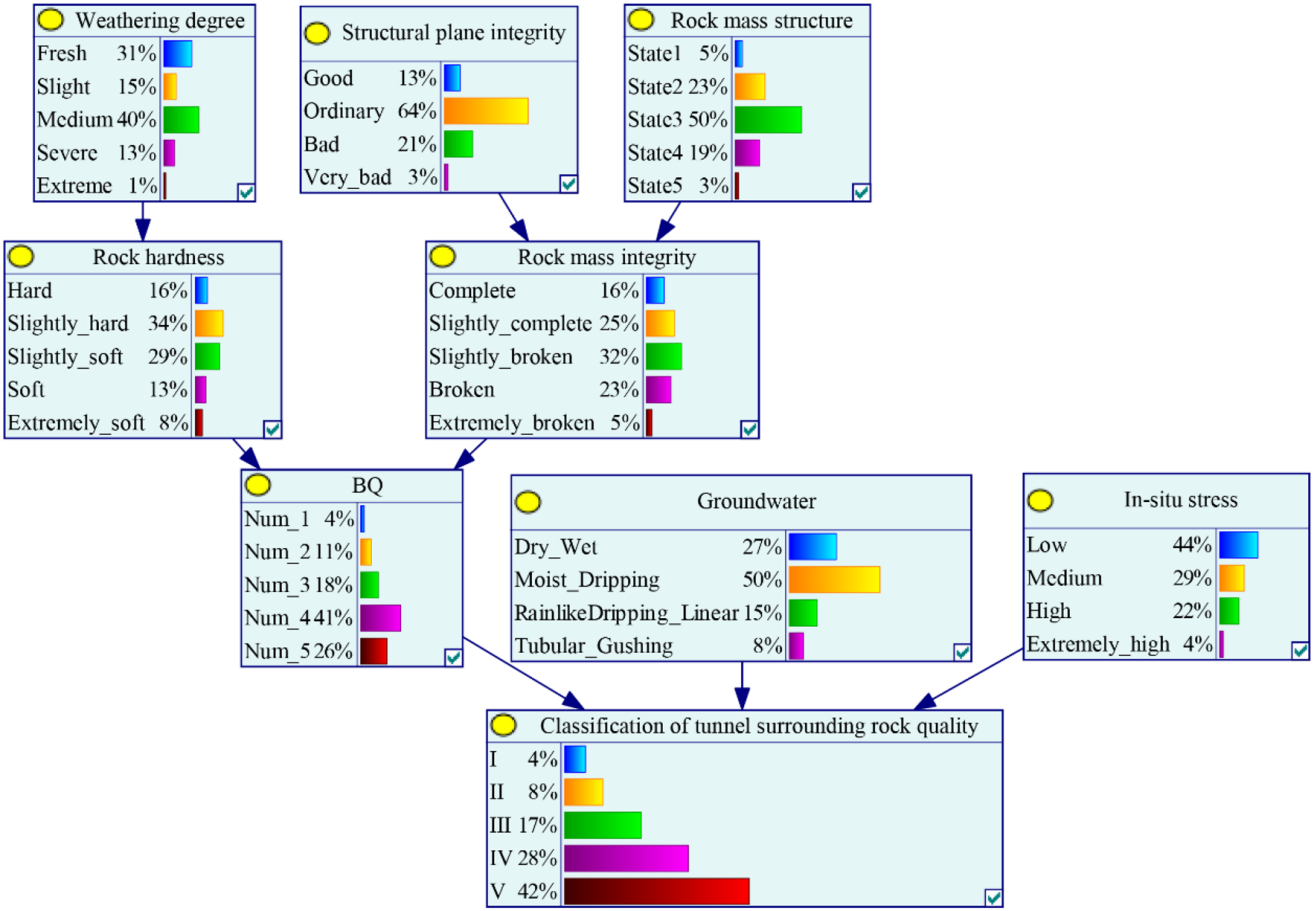
SRC-BN model after parameter learning using EM algorithm. State1, state2, state3, state4, and state5 of rock mass structure represent integral/extremely-thick layered, block/thick layered, fractured block/medium-thick layered/interlayer/thin layered/mosaic, fragmented, and granular, respectively.

Based on the collected 286 cases for the classification of tunnel surrounding rock quality cases, we use the GeNIe software to train the SRC-BN model. The SRC-BN structure is established by combining expert background knowledge and Bayesian search algorithm. The EM algorithm is used to learn the network parameters, and the JT algorithm is used to update beliefs to convey the uncertainty (before parameter learning, we assume that there is no prior knowledge, so the prior probability of each state of the node is the same). In addition, the learned CPTs in the SRC-BN model are also evaluated and revised based on the expert experience, which improves the model performance in the case of small and imbalanced samples. The trained SRC-BN model is shown in Fig. 8. Due to the complex SRC-BN structure, the CPTs of each node are listed in Appendix B.

After learning the structure and parameters of SRC-BN model, when a set of new observation values for the input variables are given, JT algorithm can be used for probabilistic inference to obtain the probability distribution of the target node state. For instance, let us assume that we obtain the following set of input data regarding the tunnel surrounding rock parameters: *W* = Medium, *R*_c_ = 25 MPa (*H* = Slightly soft), *D* = Ordinary, *S* = Medium-thick layered structure (State 3), *K*_v_ = 0.40 (*C* = Slightly broken), *G* = Wet, *R*_c_/*σ*_max_ = 3 (*I* = Low). Using these variable values for the predictive classification of tunnel surrounding rock quality with the trained SRC-BN model, the probability of nodes *B* and *L* are updated, and we obtain that *P* (*B* = 4|*W* = Medium, *H* = Slightly soft, *D* = Ordinary, *S* = State 3, *C* = Slightly broken) = 97.6%, and *P* (*L* = IV|*W* = Medium, *H* = Slightly soft, *D* = Ordinary, *S* = State 3, *C* = Slightly broken, *G* = Wet, *I* = Low) = 86.5%. This implies that IV is the most likely grade for such tunnel surrounding rock. SRC-BN model can also make prediction with incomplete input data. For example, *H*, *C*, *B*, and *I* are not known in the previous example, and we can recalculate the probability of the target node under the given input parameters as *P* (*L* = IV|*W* = Medium, *D* = Ordinary, *S* = State 3, *G* = Wet) = 42.9% (the probability of IV is also the largest among all states of the node). The estimated change in the probability of tunnel surrounding rock quality grade has practical significance for tunnel support and design. In addition, the results of probability of tunnel surrounding rock quality grade vary with our knowledge of the input parameters.

The predicted probability can also be manually calculated using CPTs (Appendix B) of each node variable in SRC-BN model. The posterior probability of *L* = IV given **X**, i.e., *P*(L = IV|**X**), is expressed as follows:

3 $$P\left( {L = {\text{IV}}\left| {\mathbf{X}} \right.} \right) = \frac{{P\left( {{\mathbf{X}}\left| {L = {\text{IV}}} \right.} \right)P(L = {\text{IV)}}}}{{P\left( {\mathbf{X}} \right)}} = \frac{{\prod\limits_{i = 1}^{n} {P\left( {x_{i} \left| {L = {\text{IV}}} \right.} \right)} }}{{\sum\limits_{{j = {\text{I,II,III,IV,V}}}} {\prod\limits_{i = 1}^{n} {P\left( {x_{i} \left| {L = j} \right.} \right)} } }}$$

where **X** = (*x*_1_, *x*_2_, …, *x*_*n*_) is the input vector, which represents the input variables (*n*_max_ = 8 in this study); *j* is the state of target node *L*, and *j* = I, II, III, IV, V.

Now, we illustrate how to use Eq. (3) to classify the quality of tunnel surrounding rock. The above example is used as the input data, i.e., **X** = (*W* = Medium, *H* = Slightly soft, *D* = Ordinary, *S* = State 3, *C* = Slightly broken, *G* = Wet, *I* = Low). Combining CPTs in Appendix B and Eq. (3), the posterior probability of *B* = 4 under given **X** is calculated as follows:

$$P\left( {B = 4\left| {\text{X}} \right.} \right) = \frac{0.405 \cdot 0.309 \cdot 0.635 \cdot 0.502 \cdot 0.42 \cdot 0.976}{{0.405 \cdot 0.309 \cdot 0.635 \cdot 0.502 \cdot 0.42 \cdot 1}} = 0.976$$

The posterior probability of *L* = IV under given **X** is calculated as follows:

$$\begin{aligned} P\left( {L = {\text{IV}}\left| {\mathbf{X}} \right.} \right) & = \frac{0.405 \cdot 0.309 \cdot 0.635 \cdot 0.502 \cdot 0.42 \cdot 0.266 \cdot 0.443}{{0.405 \cdot 0.309 \cdot 0.635 \cdot 0.502 \cdot 0.42 \cdot 0.266 \cdot 0.443 \cdot 1}} \\ & \quad \cdot \frac{{0.006 \cdot \left( {0.025 + 0.015 + 0.029 + 0.029} \right) + 0.976 \cdot 0.886}}{0.006 \cdot 4 + 0.976} = 0.865 \\ \end{aligned}$$

The result of manual calculation is the same as that of GeNIe. Thus, based on Eq. (3) and CPTs in Appendix B, the classification-probability of tunnel surrounding rock quality can be easily predicted without any software.

### SRC-BN model validation

#### Ten-fold cross-validation

**Figure 9:**
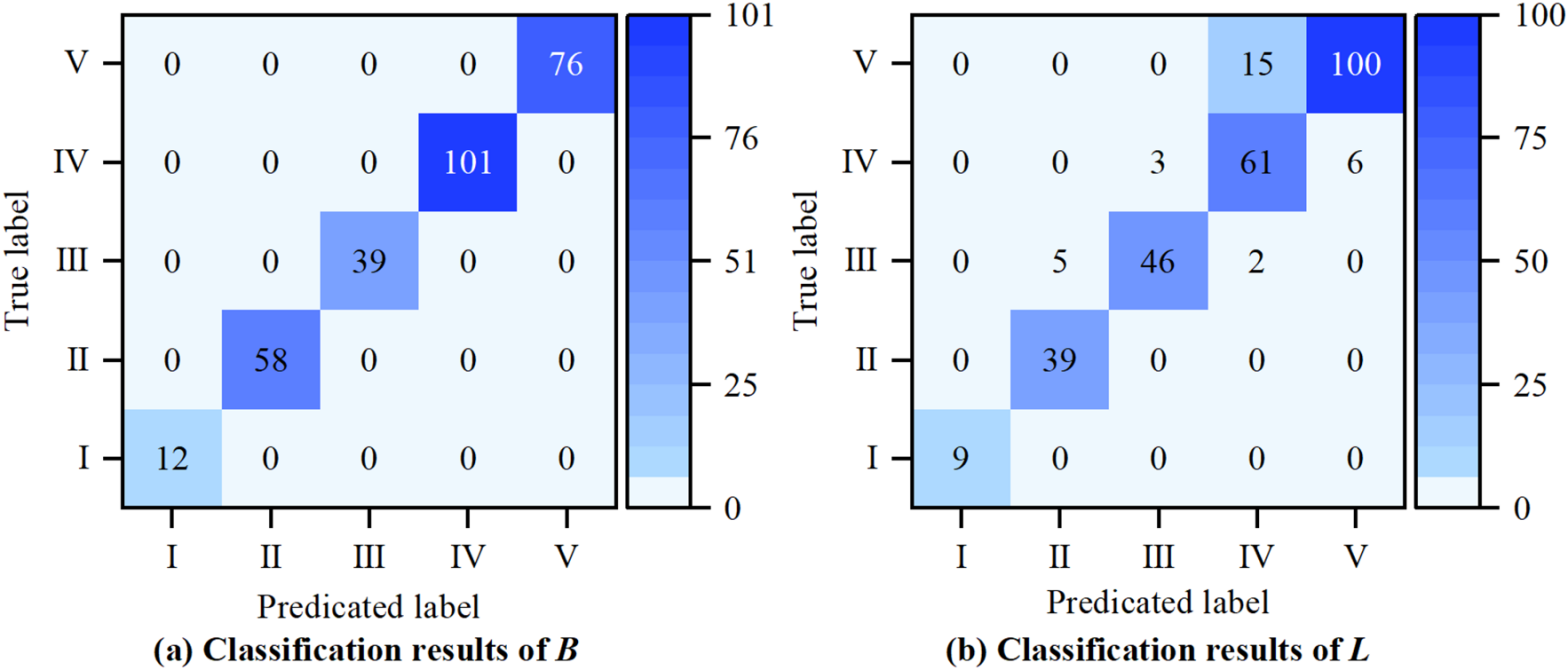
Confusion matrix of ten-fold cross-validation.

**Figure 10:**
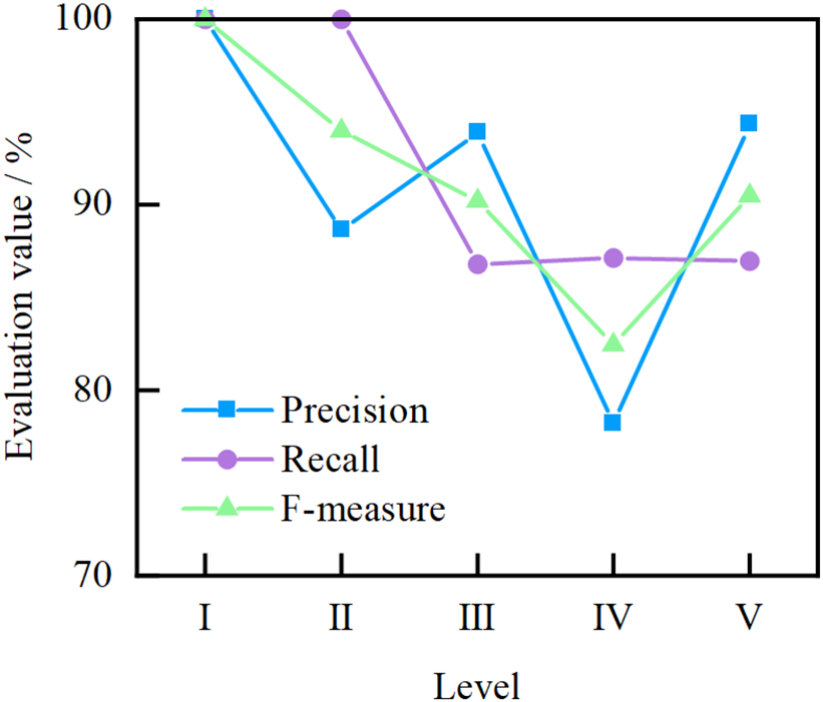
Precision, recall, and F-measure for classification variable *L.*

**Figure 11:**
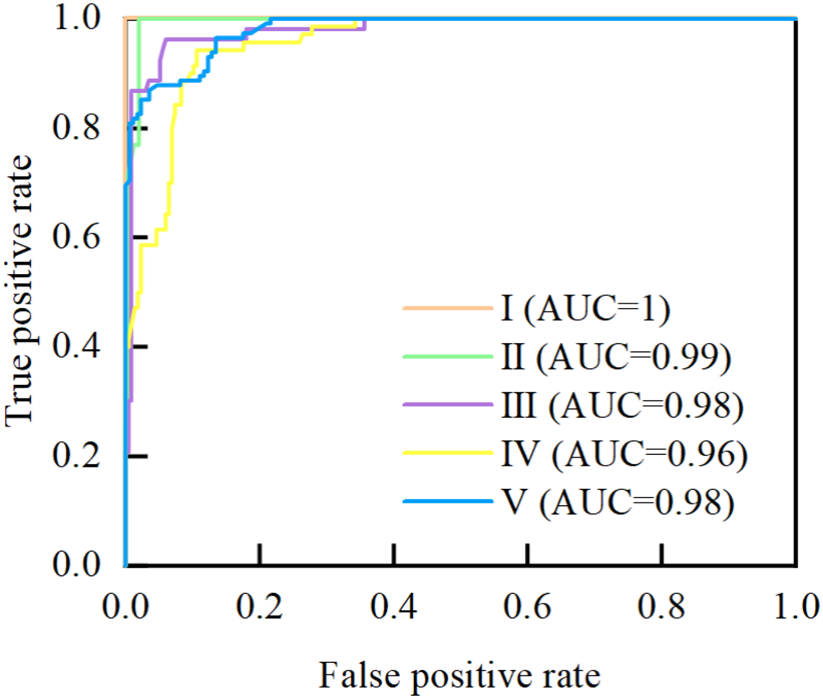
ROC curves for classification variable *L.*

According to the influencing factors of surrounding rock quality, it can be known that the corresponding samples are inherently imbalanced. Therefore, the ten-fold cross-validation method is used to validate the proposed model, and the accuracy, precision, recall, F-measure, and ROC curves are used to evaluate the performance of the model. The details of model evaluation methods and indicators are provided in Appendix C. Firstly, all the data (286 sets) are randomly divided into 10 groups. Then, nine groups are used as training sets, and the remaining one group is used as the verification set. Finally, the process is repeated 10 times to maximize the use of each set of data to train and validate the model. The average accuracy of the 10 validation sets is used as the performance measure of the model. In the established SRC-BN model, *B* and *L* are used as the classification variables, so the average accuracy of the established model for these variables is measured in the model test. The results of ten-fold cross-validation are shown in Fig. 9. The values on the diagonal of the heatmap in Fig. 9 indicate the number of correct predictions. It is clear that the average accuracies of the model for classification variables *B* and *L* are 100% and 89.2%, respectively. Since the accuracy of variable *B* is 100%, only the prediction of the learned model for variable *L* is analyzed in detail. It can be seen in Fig. 10 that the prediction precision and recall of the learned model for each sub-state of the variable *L* are both above 85%, except the state IV, whose prediction precision is approximately 78%. Then, the learned model’s F-measure for each sub-state of the variable *L* is greater than 90%, except that for the state IV is nearly 82%. The average values of precision, recall and F-measure are approximately 91%, 92%, and 91%, respectively. Generally, the closer the precision, recall, and F-measure to 100%, the better the learned model. In addition, Fig. 11 shows that the area under the curve (AUC) of the learned model for each sub-state of the variable *L* is close to 1. The average value of AUC is 0.98. This verifies the feasibility of the proposed model for predicting the classification-probability of surrounding rock quality, even under a small and imbalanced database.

#### Validation with new samples

To further verify the effectiveness of the learned SRC-BN model, the *Q* and *RMR* methods are used to test 10 cases from three underground engineering projects, including hydropower and railway tunnel projects. Because the *Q* and *RMR* methods are inconsistent with the grading standards used in this article, according to previous studies^64–67^, the *Q* and *RMR* grades are standardized (see Table 11). The input parameters and quality classification results of surrounding rock related to the proposed SRC-BN model are shown in Table 12. It is worth noting that the *Q* and *RMR* value scoring in Table 12 are based on the existing literature, and not on this study. In addition, all the 10 test cases do not include the rock mass integrity parameter, and some test cases lack the groundwater and in-situ stress parameters. The above learned SRC-BN model is capable of handling incomplete data.

**Table 11:** Relationship between [*BQ*], *Q*, and *RMR* rock mass classification methods.

Methods	I	II	III	IV	V
[*BQ*]	(550, 710]	(450, 550]	(350, 450]	(250, 350]	(0, 250]
*Q*	[100, 1000]	[10, 100)	[1, 10)	[0.1, 1)	[0.001, 0.1)
*RMR*	[70, 100]	[60, 70)	[40, 60)	[20, 40)	[0, 20)

**Table 12:** Verification results of the new tunnel cases.

No	Project	*W*	*H* (MPa)	*D*	*S*	*B*	*G*	*I*	BN	*Q*	*RMR*	Actual
1	Baihetan hydropower station PD62^65^	Medium	60	Good	Fractured block	3	Dry	Medium	84.0% III	3.65 III	61 II	III
2		Medium	60	Good	Fractured block	3	Wet	Medium	84.0% III	1.36 III	66 II	III
3		Medium	75	Good	Block	2	Wet	Medium	60.0% II	42.95 II	63 II	II
4		Medium	70	Good	Fractured block	2	Wet	Medium	60.0% II	173.2 I	68 II	II
5		Slight	100	Good	Fractured block	2	Wet	Medium	60.0% II	56.8 II	72 I	II
6		Slight	100	Good	Block	2	wet	Medium	60.0% II	114 I	70 II	II
7	Lenggu hydropower station CPD01^68^	Slight	–	Ordinary	Fractured block	3	–	–	63.8% III	1.9 III	45 III	III
8		Slight	–	Good	Mosaic structure	3	–	–	63.8% III	3.1 III	43 III	III
9		Slight	–	Ordinary	Fractured block	3	–	–	63.8% III	1.1 III	38 IV	IV
10	Duncun tunnel^69^	Severe	15	–	Fragmented structure	5	Moist	Medium	90.9% V	0.06 V	–	V

The "Actual" in the last column of the table is the quality grade of surrounding rock referenced by the actual support at the tunnel construction site.

**Figure 12:**
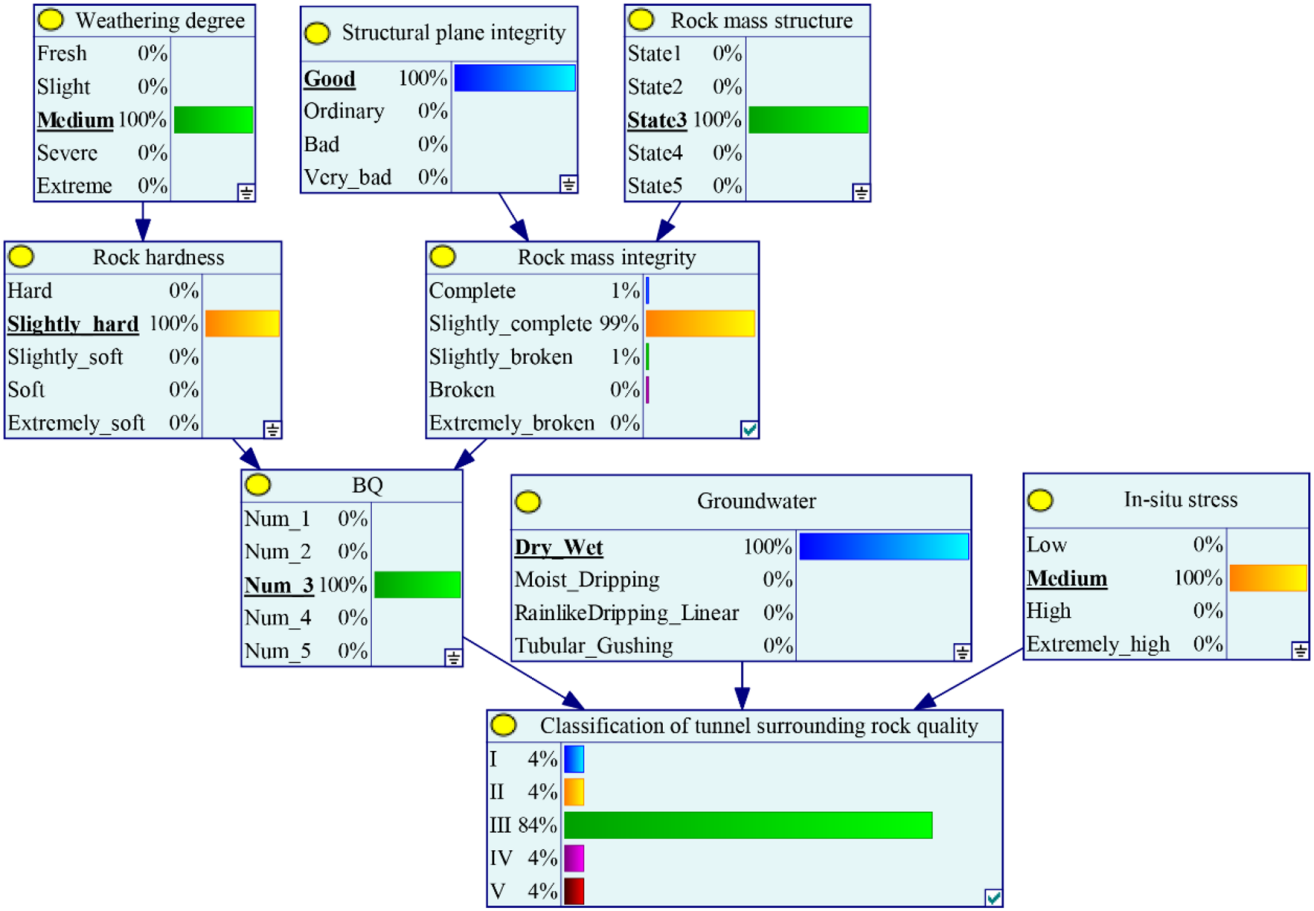
Posterior probability of target nodes in GeNIe.

The classification no. 1 in Table 12 is taken as an example to calculate the posterior probability of variable nodes through GeNIe (see Fig. 12). The nodes with a probability of 100% in Fig. 12 are known evidence, and the posterior probability distribution of nodes with unknown evidence can be obtained by calculation. As shown in Fig. 12, the probability of a slightly complete rock mass is approximately 98.7%, and that of grade III surrounding rock is nearly 84.0%.

It can be seen from Table 12 that the BN method predicts one case (case 9) incorrectly, which has more missing data, so the prediction results are conservative. In addition, the *Q* and *RMR* methods predicts three error cases. According to the surrounding rock quality classification results in Table 12, although the accuracy of the established SRC-BN model is not much different from the internationally popular *Q* and *RMR* methods, it has a wider application scope (especially with incomplete data).

### Sensitivity analysis

**Figure 13:**
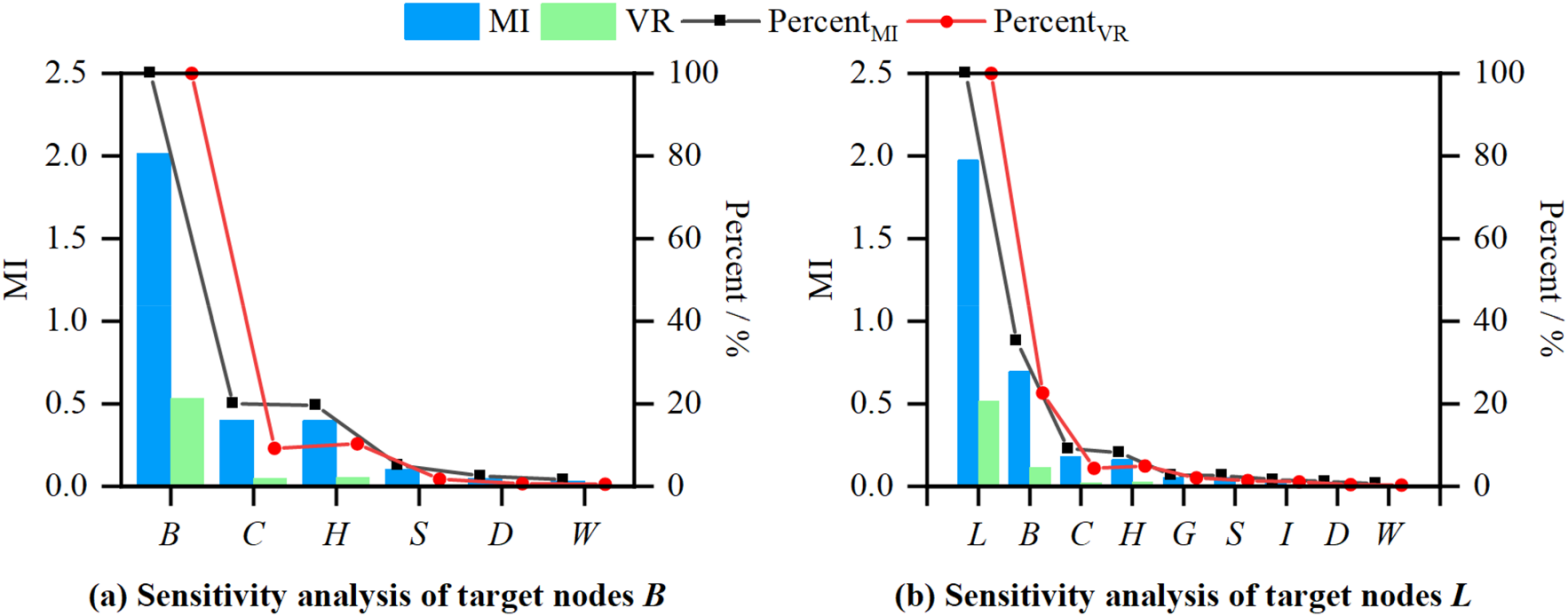
Results of sensitivity analysis based on the SRC-BN model.

Since the SRC-BN structure is a tree structure, it is not a radial structure that conforms to the naive Bayesian theory (the input variables are independent of each other and are only related to the target node). Therefore, sensitivity analysis is used to determine which variables contribute the most to the prediction of surrounding rock quality levels. Mutual information (MI) and variance reduction (VR) are employed to measure the parametric sensitivity. The specific measurement indicators can be found in Shannon^70^ and Pearl^71^. Fig. 13 shows the sensitivity analysis results calculated by Netica, including the influence of five input parameters on *BQ* (*B*) and the influence of eight input parameters on the classification of surrounding rock quality (*L*). Percent represents the contribution of each input variable to the target node. Here, nodes *B* and *L* are set as the target node. It is observed that the rock mass integrity (*C*) and rock hardness (*H*) have the highest contribution to the *BQ* (*B*). The *B* has the highest contribution to the *L*. Therefore, the hardness and integrity of rock mass have a greater impact on the final judgment of the surrounding rock quality. These results and parameter description in “Inputs of SRC-BN” are consistent with the strength analysis in “Strength analysis”. Of course, other parameters (*W*, *D*, *S*, *G*, and *I*) are also important for predicting the classification of surrounding rock quality. Since the SRC-BN model has a tree structure (non-naive Bayesian structure), the calculated sensitivity value of the node that is not directly connected to the target node is small. Although the mutual information and variance reduction in Fig. 13 are relatively small, their influence on the classification of surrounding rock quality should also be taken into account.

## Conclusion

In this study, we proposed a new method based on BNs to predict the classification-probability of tunnel surrounding rock quality with incomplete information. The intelligent SRC model that can handle incomplete data is of great significance for the dynamic design and construction of tunnels. The main findings of this study can be summarized as follows.

1. A probabilistic prediction model of surrounding rock quality grades was established through structural learning and parameter learning based on 286 sets of cases. Strength analysis was utilized to quantitatively verify the rationality of the learned SRC-BN structure.
2. The ten-fold cross-validation results showed that the accuracy of categorical variables *B* and *L* was 100% and 89.2%, respectively. The average precision, recall, and F-measure values for variable *L* were over 91%. The AUC for each sub-state of variable *L* exceeded 0.96. The evaluation results verified the stable and good performance of the learned model for predicting the classification-probability of surrounding rock quality, even with a small and imbalanced database.
3. The validation results with ten samples suggested that the error rate of the learned SRC-BN model was lower than that *Q* and *RMR* methods. The results indicated that the learned SRC-BN model could identify the surrounding rock grade more accurately and intelligently, even when the original information was incomplete.
4. The sensitivity analysis results showed that the hardness and integrity of the rock mass were the main parameters that affected the quality of tunnel surrounding rock. Obviously, other parameters affected the quality of tunnel surrounding rock to a certain extent, among which the weathering degree had the lowest impact.

Overall, the proposed SRC-BN model can probabilistically predict the quality grade of tunnel surrounding rocks with incomplete data, which has great application value in the tunnel and underground engineering. Moreover, the results also have certain reference value for other rock mass engineering applications related to rock mass quality, such as slope engineering. However, since the training samples and verification projects of the SRC-BN model are mainly tunnels and underground projects in Southwest China, validating the applicability of the model for tunnels and underground projects in other regions needs further study. Therefore, in the future, we will collect more samples to improve the model's performance. In addition, cloud technology may be integrated to establish a cloud platform for tunnel information management.

## Supplementary Information

Supplementary Information 1.

Supplementary Information 2.

Supplementary Information 3.

## Data Availability

The datasets generated or analyzed during the current study are available from the corresponding author upon reasonable request.

## List of symbols

*R*_c_: Saturated uniaxial compressive strength
*k*_*r*_: Wave velocity ratio
*k*_*f*_: Weathering coefficient of rock
*K*_v_: Rock mass integrity coefficient
*V*_pm_: Elastic longitudinal wave velocity of rock mass
*V*_pr_: Elastic longitudinal wave velocity of rock (block)
*J*_v_: Volume joint number of rock mass
*σ*_max_: Maximum initial stress perpendicular to the hole axis
*p*: Water pressure of surrounding rock fissures
*q*: Water output per 10 m hole length
*W*: Weathering degree
*D*: Structural plane integrity
*S*: Rock mass structure
*H*: Rock hardness
*C*: Rock mass integrity
*I*: In-situ stress
*B*: Basic quality of rock mass (*BQ*)
*G*: Groundwater
*L*: Surrounding rock level
*A*: A hypothetical space point
*T*: A hypothetical space point
*K*_1_: Correction factors for groundwater
*K*_2_: Correction factors for most unfavorable structural plane attitude
*K*_3_: Correction factors for initial geo-stress field
*TP*: True positive
*FN*: False negative
*FP*: False positive
*TN*: True negative
