# Supplementary material for: A probability prediction method for the classification of surrounding rock quality of tunnels with incomplete data using Bayesian networks
Source: Sci Rep. 2022 Nov 18;12:19846. doi: 10.1038/s41598-022-19301-6 (PMC9674632; doi:10.1038/s41598-022-19301-6)
Supplement: Supplementary file 1 — Supplementary Information 1. [file 41598_2022_19301_MOESM1_ESM.docx]

Appendix A: Standard for Engineering Classification of Rock Mass in China—[*BQ*] method (GB/T 50218 - 2014)

The [*BQ*]method includes two steps to calculate rock mass quality. Then the rock mass quality is divided into five grades according to the classification table, as shown in Table A. 1.

| Rock mass level | Rock mass characteristic | Rating |
| --- | --- | --- |
| Ⅰ | Very hard, very intact | (550, 700] |
| Ⅱ | Very hard, intact  Hard, very intact | (450, 550] |
| Ⅲ | Very hard, crushed  Hard, intact  Soft, very intact | (350, 450] |
| Ⅳ | Very hard, very crushed  Hard, crushed-very crushed  Soft, intact-crushed  Very soft, very intact-intact | (250, 350] |
| Ⅴ | Soft, very crushed  Very soft, crushed-very crushed  Decomposed | (0, 250] |

Table A. 1. Engineering rock mass basic quality classification based on the [*BQ*] method

Firstly, two geological parameters representing the strength and integrity of the rock mass are used to calculate the basic quality (*BQ*) of rock mass, as shown in Eq. .

$$BQ=90+3R_{\text{c}}+250K_{\text{v}}$$

where *R*_c_ (unit: MPa) is the uniaxial compression strength of intact rock, which can be obtained by uniaxial compression test or converted by point load strength. *K*_v_ is the integrity of the rock mass. It calculated by the square of the ratio of the elastic longitudinal wave velocity of the rock mass to that of the rock.

When *R*_c_ and *K*_v_ cannot be obtained by actual measurement, they can be determined through the qualitative scheme given by *Standard for Engineering Classification of Rock Mass* (not listed here due to length limit).

Secondly, considering the effects of groundwater, weak structural plane, and initial geo-stress field, the *BQ* of rock mass is further modified to obtain [*BQ*] (Eq. ).

$$\left[BQ\right]=BQ-100\left(K_{1}+K_{2}+K_{3}\right)$$

where *K*_1_, *K*_2_, and *K*_3_ are the correction factors for groundwater, most unfavorable structural plane attitude, and initial geo-stress field, respectively (the specific recommended values are obtained from the *Standard for Engineering Classification of Rock Mass*).

Finally, the rock mass level is determined by comparing the [*BQ*] value with the grade in Table A. 1.
