# Supplementary material for: A probability prediction method for the classification of surrounding rock quality of tunnels with incomplete data using Bayesian networks
Source: Sci Rep. 2022 Nov 18;12:19846. doi: 10.1038/s41598-022-19301-6 (PMC9674632; doi:10.1038/s41598-022-19301-6)
Supplement: Supplementary file 3 — Supplementary Information 3. [file 41598_2022_19301_MOESM3_ESM.docx]

Appendix C: Ten-fold cross-validation

The flow diagram of ten-fold cross-validation is shown in Fig. C. 1. First, the data is divided into 10 equal parts (N1-N10). Then, one of the ten groups is sequentially selected as the validation set, and the remaining nine groups are considered as the training set. This cross-validation is repeated ten times. Finally, the results of 10 verifications are averaged to evaluate the performance of the model.


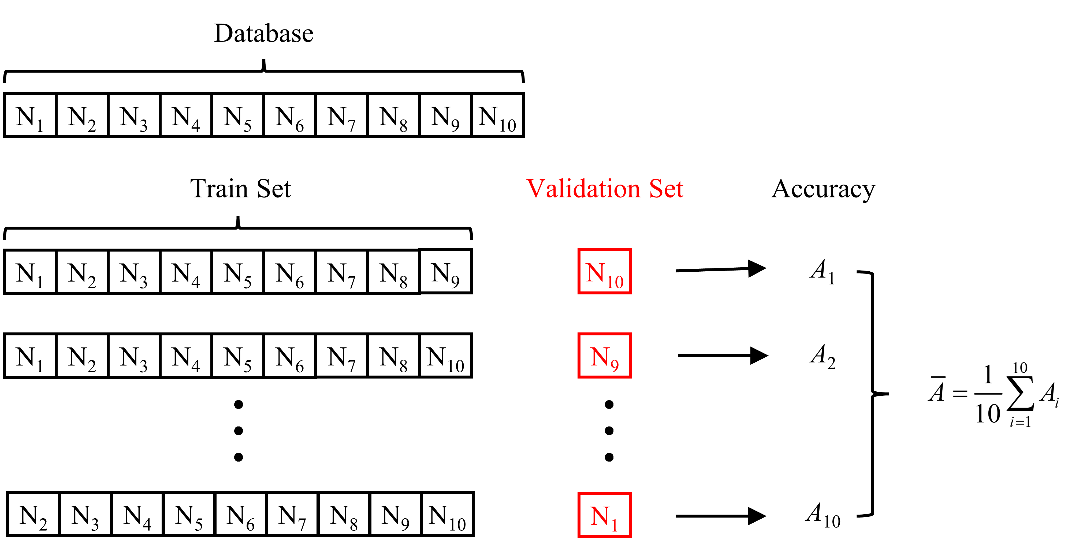


Figure. C. 1. Schematic diagram of ten-fold cross-validation

A variety of indicators must be used to evaluate the performance of the model under the condition of imbalanced data, such as accuracy, precision, recall, F-measure, and receiver operating characteristic (ROC) curves. A binary classifier is assumed, where the classification status is true and false. The confusion matrix of the prediction result is shown in Table C. 1.

| -- | | Predicted | |
| --- | --- | --- | --- |
|  |  | True | False |
| Actual | True | True Positive (*TP*) | False Negative (*FN*) |
|  | False | False Positive (*FP*) | True Negative (*TN*) |

Table C. 1. Confusion matrix

Accuracy is the proportion of correct judgment (Eq. ). Precision is the ratio of the data that is predicted to be true to the data that is actually true (Eq. ). Recall is the ratio of the number of true positives to the sum of number of true positives and number of false negatives (Eq. ). F-measure is the harmonic average of precision and recall (Eq. ). In fact, the closer the values of the above four indicators to 1, the better the model performance.

ROC curves reflect the stability of the model when the class of the dataset is imbalanced. The ROC curve is analyzed by calculating the area under the curve (AUC). The larger the AUC, the better the model. As shown in Fig. C. 2, the red line along the coordinate axis is the ROC curve of the best model. The dashed line is the ROC curve of invalid model. When the ROC curve is below the dotted line, the performance of the model is poor. The area of the shaded part in the Fig. C. 2 is the AUC of the ROC curve.


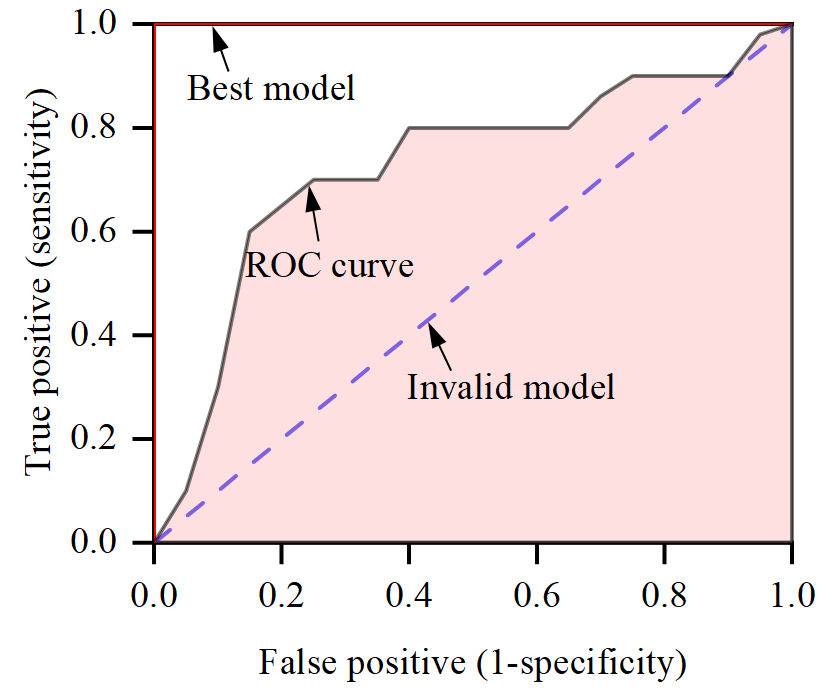


Figure. C. 2. ROC curves
